# Dissecting genetic architecture of startle response in *Drosophila melanogaster* using multi-omics information

**DOI:** 10.1038/s41598-017-11676-1

**Published:** 2017-09-28

**Authors:** Angli Xue, Hongcheng Wang, Jun Zhu

**Affiliations:** Institute of Bioinformatics, Zhejiang University, Hangzhou, 310058 China

## Abstract

Startle behavior is important for survival, and abnormal startle responses are related to several neurological diseases. *Drosophila melanogaster* provides a powerful system to investigate the genetic underpinnings of variation in startle behavior. Since mechanically induced, startle responses and environmental conditions can be readily quantified and precisely controlled. The 156 wild-derived fully sequenced lines of the *Drosophila* Genetic Reference Panel (DGRP) were used to identify SNPs and transcripts associated with variation in startle behavior. The results validated highly significant effects of 33 quantitative trait SNPs (QTSs) and 81 quantitative trait transcripts (QTTs) directly associated with phenotypic variation of startle response. We also detected QTT variation controlled by 20 QTSs (tQTSs) and 73 transcripts (tQTTs). Association mapping based on genomic and transcriptomic data enabled us to construct a complex genetic network that underlies variation in startle behavior. Based on principles of evolutionary conservation, human orthologous genes could be superimposed on this network. This study provided both genetic and biological insights into the variation of startle response behavior of *Drosophila melanogaster*, and highlighted the importance of genetic network to understand the genetic architecture of complex traits.

## Introduction

With the diminishing cost of high-throughput technologies, including rapid genome-sequencing methods and whole genome transcript profiling, the focus of genomic sciences is shifting from data production to data interpretation^1^. Genome-wide association studies (GWASs), which examine genetic variants across the genome by associations with variation in traits^2^, can reveal the extent of functional genetic variation in natural populations. There remains, however, a substantial gap in our understanding of how the concerted effects of SNPs across the genome relate to phenotypic variation. In addition to GWAS, gene expression levels have been used as quantitative traits for eQTL mapping to DNA markers^3^. Such studies enable us to categorize *cis* and *trans* effects of SNPs on gene expression. Thus, eQTL analysis is often incorporated as a component of GWAS to support causality of candidate genes.

An ever-increasing number of human disease genes will be identified in the coming years along with a deeper understanding of the genetic risk factors for complex diseases. Genetic studies of human diseases have limitations, due to uncontrolled genetic backgrounds, diverse environmental exposures, difficulty to precisely quantify phenotypes, especially for behavioral traits, and the extent of linkage disequilibrium within the human genome^4^. *Drosophila melanogaster* is able to serve as a valuable model to assess the genetic architecture of complex traits and, based on evolutionary conservation of fundamental principles, insights from this model system can be applied to human quantitative traits and diseases^5^. To date, GWASs have been conducted for both organismal and gene-expression traits using wild-derived inbred fly lines with fully sequenced genomes^6^. Numerous loci identified in these studies are novel, including predicted transcripts of unknown function. In addition, sex- and environment-specific effects and epistasis are prominent in the genetic architecture of many complex traits, such as chill coma recovery and starvation resistance^4,7,8^. Many genes identified by GWASs to date play roles in early development, while others are computationally predicted without annotation^9^. System genetic approaches that combine analyses of variation at the DNA level with transcriptional variation and variation in organismal phenotype allow the construction of gene networks associated with complex traits in *Drosophila* ^6,10^.

Although GWASs have identified hundreds of common variants associated with complex traits and susceptibility loci have been reported for many diseases, the overall genetic risk explained by these loci remains modest^11^. This could be attributed to a preponderance of a vast number of rare alleles that evade detection even with large sample sizes. Here, we present analyses of variation in startle response, a universal survival behavior, to investigate fundamental features of the genotype-phenotype relationship that underlies the manifestation of complex traits.

A simple, high-throughput and reproducible assay to quantify startle-induced locomotion has been developed and used to map candidate genes corresponding to QTLs. Transcriptional analysis indicated that a large fraction of the genome affects this trait^12^ and a previous study showed evidence for epistasis both at the level of QTLs and *P*-element-induced mutations^13^. As might be expected, genes required for nervous system development were implicated for startle-induced locomotion^14^. An analysis of epistatic interactions among a subset of these genes showed a network of genes associated with neurodevelopment^12^.

As an important feature of the genetic architecture of complex traits, epistasis can generate novel phenotypes^15^, and is difficult to be detected both in genome scans and experiments^13^. To detect pairwise or higher-order interactions, powerful computational capability and appropriate statistical approaches are needed^16^. Epistasis was indicated in various of traits of *Drosophila*, such as aggressive behavior^17^. Since some genes are differentially expressed between males and females, the sex-biased expression is a potential mechanism that can maintain genetic variation during the course of evolution^6^. Sex-specific genetic architecture is quite common and natural variations in the autosome can also affect traits differentially in males and females in many species^18^. Although the contributions of genes to a phenotype can often be well established, little is known about the regulation and interactions of these genes^19^. Taking into account regulatory networks will provide a better understanding of the genetic mechanisms that orchestrate complex traits^20^.

Here, we combined analyses of SNPs and transcripts to elucidate genetic networks associated with startle behavior in *Drosophila*, which took into account gene-gene interactions and sex-specific effects. Previous studies focused on SNPs or transcripts association with phenotypes, and we firstly integrated QTSs and QTTs for network analysis in *Drosophila*. We found that several QTSs were associated with QTTs, and some QTTs were associated with other QTTs, which provided more information of the regulatory process of startle response.

## Materials and Methods

We downloaded the published genotype data of 156 inbred lines from the *Drosophila melanogaster* Genetic Reference Panel (DGRP)^21^. The transcriptome data of 156 lines were also downloaded from the Mackay Laboratory (http://dgrp2.gnets.ncsu.edu/)^22^. All these lines were derived from inbred lines by the Raleigh, NC, USA population for 20 generations of full-sib mating to produce viable, fertile inbred lines^21^. Transcript data were obtained from 3- to 5-day-old mated males and females for each of DGRP lines by Affymetrix Drosophila 2.0. All samples were frozen between 1 and 3 pm. The RNAs were extracted from two independent pools (25 flies/sex/line), and hybridized to 10 µg fragmented cRNA to each array^21^. Startle-induced locomotion was quantified as the number of seconds each fly was active in the 45 s period immediately following the disturbance. There were two replicated measurements (20 flies/sex/replicate) per line. The replicates for each line were assessed on different days. All flies were reared and all phenotypes assessed under standard culture conditions (cornmeal-molasses-agar-medium, 25 °C, 60~75% relative humidity, 12-h light-dark cycle) unless otherwise specified. The sample size for all experiments was *N* = 20 measurements/sex/genotype^13^.

### Mixed linear model

We used a mixed linear model to detect individual and epistatic loci of SNPs or transcripts significantly associated with variation in startle behavior in the DGRP lines. The genetic model for phenotypic value of the *k-*th genotypes in the *h-*th gender (*y*_hk_) can be expressed by the following mixed linear model,

1 $${y}_{kh}=\mu +\sum _{i}{q}_{i}{\mu }_{ik}+\sum _{i < j}q{q}_{ij}{u}_{ijk}+{e}_{h}+\sum _{i}q{e}_{ih}{\mu }_{ikh}+\sum _{i < j}qq{e}_{ijh}{u}_{ijkh}+{\varepsilon }_{kh}$$

where *μ* is the population mean; *qi* is the *i-*th locus effect for both genders with coefficient *u*_*ik*_; *qq*_*ij*_ is the epistasis effect of the *i-*th locus by *j-*th locus for both genders with coefficient; *u*_*ik*_; *e*_*h*_ is the effect of the *h-*th gender (*e*_*1*_ = male, *e*_2_ = female); *qe*_ih_ is the locus by gender interaction effect of the *i-*th locus in the *h-*th gender with coefficient *u*_*ikh*_; *qqe* is the epistasis-sex interaction effect of the *i-*th locus by *j-*th locus in the *h-*th sex with coefficient *u*_*ijkh*_; and ε_*kh*_ is the random residual effect of the *k-*th breeding line in the *h-*th gender.

This genetic model can be used in association mapping by setting phenotype traits as dependent variants **y** for association to independent variants of quantitative trait SNPs (QTSs) or of quantitative trait transcripts (QTTs), and also by setting transcript abundance levels as dependent variants **y** for association to independent variants of SNPs or of transcripts.

### Estimation of genetic effects and heritability

Association analyses were conducted for detecting SNPs by QTS modular and transcripts by QTT modular using mixed linear model approaches^23^. Genetic effects were estimated by software *QTXNetwork* (http://ibi.zju.edu.cn/software/QTXNetwork/) based on GPU parallel computation^24^. The phenotypic variance (***V***_p_) is the sum of genotype variance (***V***_*G*_), genotype by environment interaction variance (*V*_*GE*_), and residual variance (*V*_*ε*_), which can be written as follows:

2 $$\begin{array}{rcl}{V}_{P} & = & {V}_{G}+{V}_{GE}+{V}_{\varepsilon }\\  & = & {V}_{Q}+{V}_{QQ}+{V}_{QE}+{V}_{QQE}+{V}_{\varepsilon }\end{array}$$

Heritability is defined as the relative contribution of genetic variance to phenotypic variance with the following estimation model:

3 $$\begin{array}{rcl}{h}_{T}^{2} & = & ({h}_{Q}^{2}+{h}_{QQ}^{2})+({h}_{QE}^{2}+{h}_{QQE}^{2})\\  & = & (\sum _{i}{h}_{q}^{2}+\sum _{i < j}{h}_{qq}^{2})+(\sum _{i}{h}_{qe}^{2}+\sum _{i}{h}_{qqe}^{2})\\  & = & (\displaystyle \frac{1}{d{f}_{q}}\sum _{i}{q}_{q}^{2}/{V}_{P}+\displaystyle \frac{1}{d{f}_{qq}}\sum _{i < j}{q}_{qq}^{2}/{V}_{P})+(\displaystyle \frac{1}{d{f}_{qe}}\sum _{i}{q}_{qe}^{2}/{V}_{P}+\displaystyle \frac{1}{d{f}_{qqe}}\sum _{i}{q}_{qqe}^{2}/{V}_{P})\end{array}$$

where ${h}_{T}^{2}$ = total heritability; ${h}_{Q}^{2}$ = heritability contributed by the sum of individual loci, ${h}_{QQ}^{2}$ = heritability contributed by the sum of pair-wise epistasis loci, ${h}_{QE}^{2}$ = gender-specific heritability contributed by the sum of individual gender-specific loci, ${h}_{QQE}^{2}$ = gender-specific epistasis heritability contributed by the sum of pair-wise epistasis loci; ${h}_{q}^{2}$ = heritability of individual locus, ${h}_{qq}^{2}$ = heritability of epistasis locus, ${h}_{qe}^{2}$ = heritability of individual locus with gender-specific effect, ${h}_{qqe}^{2}$ = heritability of individual epistasis locus with gender-specific effects.

### Association analyses

The set of SNPs that was analyzed contained no missing data and all calls were homozygous. SNPs were used with coverage greater than 2X but less than 30X, for which the minor allele was presented in at least four lines, and for which SNPs were called in at least 60 lines. The few SNPs on the very short chromosome 4 were not considered. We used 5,212,611 SNPs for mapping QTSs in 156 DGRP lines for genders separately. A two-step strategy was applied in this analysis. First, we tested the significance of the *i*-th individual locus using Equation 1, and 4,329 autosomal SNPs were identified after a scan for significant candidate SNP markers (*P*-value < 0.001) by generalized multifactor dimensionality reduction (*GMDR*)^25^. Subsequently, mixed linear model approaches in *QTXNetwork* were applied for additional screening and identification of significant SNPs by setting a total of 2,000 permutation tests to calculate the critical *F*-value to control the experiment-wise type I error (${\alpha }_{EW}\le 0.05$). The QTS effects were estimated using the Monte Carlo Markov Chain method with 20,000 Gibbs sampler iterations^23^. Second, after selecting the 4,329 candidate loci, a full statistical model as described in Equation 1 was applied to estimate variance components and genetic effects by mixed linear model approaches.

We also analyzed 10,122 genetically variable transcripts for 156 DGRP lines, to identify transcripts of which variation in expression was associated with variation in startle behavior. Epistasis interactions were accessed among associated SNPs and among transcripts of which variation in expression correlated with variation in startle response using *QTXNetwork* by the same approach as for detecting significant QTSs.

## Results

There were 48 QTSs significantly associated with startle response, among which 33 QTSs were experiment-wise highly significant (−Log_10_ *P*_*EW*_ ≥ 5). The total heritability (${h}_{T}^{2}$ $\hat{=}$ 93.48%) was composed of additive heritability (${h}_{A}^{2}$ $\hat{=}$ 83.62%) and epistasis heritability (${h}_{AA}^{2}$ $\hat{=}$ 10.86%) (Table 1). Highly significant QTSs for startle behavior were presented in Table S1. Two SNPs, 2R_1441701_T and 2L_12568798_C, had large additive effects with high heritability. Three pairs of epistasis effects were detected. Among 33 highly significant SNPs, most had relatively small heritability. More than half of the SNPs were located in genes, while others were intergenic. The determination coefficient between total predicted genotypic effects of QTSs and phenotypic values of startle response (${R}_{\hat{G}\mapsto Y}^{2}\hat{=}0.949$) were very close to the total heritability, indicating high reliable for predicting startle response by using genetic effects of detected QTSs. Association was also conducted between transcripts and phenotypic variation of startle response for 156 DGRP lines (Table S2). There were 85 significant QTTs detected with no epistasis interactions. The total heritability (${h}_{T}^{2}\,\hat{=}$ 0.996) was mostly composed of main effects for both genders ( 0.991) (Table 1). The determination coefficient between total predicted genotypic effects of QTTs and phenotypic values of startle response were also very close to the total heritability (${R}_{\hat{G}\mapsto Y}^{2}\hat{=}0.949$), indicating high reliable for predicting startle response by using genetic effects of associated QTTs.

**Table 1:** Heritability and determination coefficient of QTSs and QTTs for startle response in *Drosophila melanogaster*.

QTS				QTT			
${h}_{A}^{2}$	${h}_{AA}^{2}$	${h}_{T}^{2}$	${R}_{\hat{G}\to Y}^{2}$	${h}_{Q}^{2}$	${h}_{QE}^{2}$	${h}_{T}^{2}$	${R}_{\hat{G}\to Y}^{2}$
0.826	0.109	0.935	0.949	0.991	0.005	0.996	0.996

${h}_{A}^{2}$ = heritability of additive effects. ${h}_{AE}^{2}$ = heritability of sex-specific additive effects. ${h}_{AA}^{2}$ = heritability of additive × additive epistasis effects. ${h}_{Q}^{2}$ = heritability of expression effects. ${h}_{Q}^{2}$ = heritability of sex-specific expression effects. ${h}_{T}^{2}$ = total heritability of all effects. ${R}_{\hat{G}\mapsto Y}^{2}$ = determination coefficient between total predicted genotypic effects and phenotypic values.

The tQTT mapping was performed to detect transcripts associated with 55 QTTs of startle response, which meant the tQTT indirectly affecting the startle trait. We identified 209 tQTTs on 55 QTTs with mean of total heritability ${h}_{T}^{2}$ $\hat{=}$ 38.75% (${h}_{T}^{2}$ $\hat{=}$ 2.08~83.39%), mostly due to main effects of individual loci (${h}_{Q}^{2}$ $\hat{=}$ 26.00%, ${h}_{Q}^{2}$ $\hat{=}$ 0.00~67.48%). There were 91 tQTTs associated with 42 QTTs at −Log_10_ *P*_*EW*_ ≥ 3 (Table S3). Gender-specific effects were detected only in tQTT level, including *XLOC_003577* to *Rab32*, *CR42765* to *XLOC_002412*, and *CR43270* to *XLOC_001023*. *XLOC_003577* and *CR43270* had both male- and female-specific effects and occurred in opposite direction, male negative and female positive, while *FBgn0085278* and *CR42765* had positive male-specific and negative female-specific effects.

We conducted tQTS mapping to detect transcripts associated with QTSs controlling startle response. (Table S4). There were 25 SNPs associated with 7 transcripts with low means of total heritability (${h}_{T}^{2}$ $\hat{=}$ 21.22%, 7.77~42.28%), mostly due to additive effects (${h}_{A}^{2}$ $\hat{=}$ 16.85) and epistatic effects (${h}_{AA}^{2}$ $\hat{=}$ 2.39). Only one pair of epistasis interactions between *CG6424* and *CG4982* was detected for *CG18067*, and we detected no sex-specific interaction. There were eight SNPs associated with variation in abundance of *CG11034*, two of which exhibited epistasis. Two SNPs were associated with variation in expression level of *frj* (essential for germ line development), which was involved in lipid modification and lysophospholipid acyltransferase activity.

Among the genes identified, five have been documented previously to affect startle-induced locomotion (*GlcAT-S*, *gukh*, *CG13196*, *l(1)G0196*, and *CG9044*)^21,26^. We compared their significance level with previous studies, and most of them were more significant (Table S5). Genetic and behavioral studies have shown that many mutations associated with reduced startle-induced locomotion played a role in nervous system development and function^12^. Gene ontology (GO) enrichment analysis showed that *glu* and *bun* played an important role in nervous system development, while *CdGAPr* and *gukh* were involved in neuron development (Table S6). The *aop* and *alpha-Spec* were both involved with these two functions. *FD64A, elk*, and *aop* were involved in regulation of transcription, while *l(2)efl* was involved in response to abiotic stimulus and stress response^27^.

**Figure 1:**
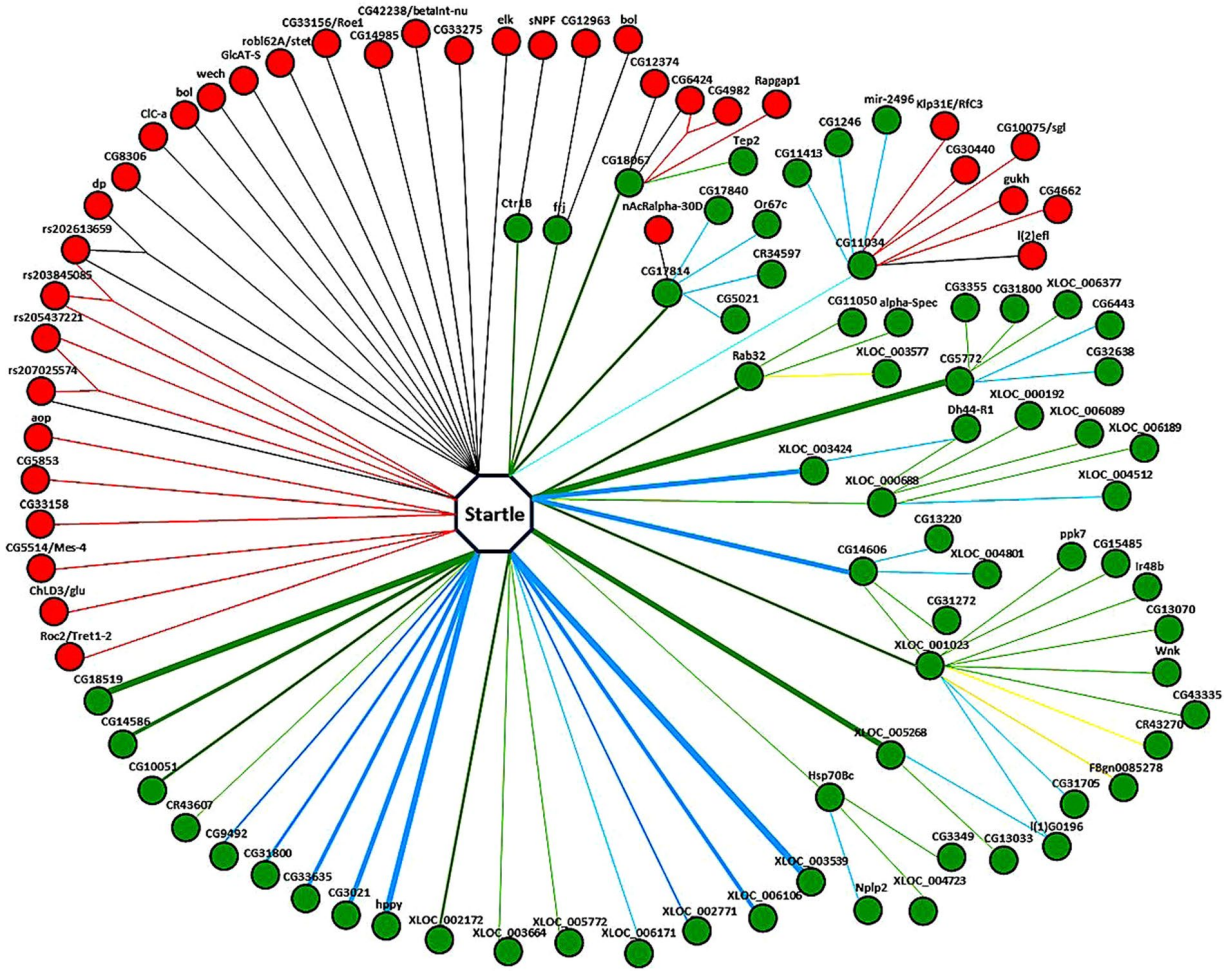
Genetic architecture of startle response controlled directly by highly significant 22 QTSs and 29 QTTs, and indirectly by 39 tQTTs and 14 tQTSs. The red nodes represent QTSs and green nodes are transcripts. The label above the node denotes the gene name. A “Y” shape line between two nodes denotes the epistasis effect. Red and green lines denote positive effects, while black and blue lines denote negative effects. Yellow lines denote positive effect in female and negative effect in male, and orange lines denote positive effect in male and negative effect in female. The width of lines denotes the value of estimate with a wider line representing a higher estimate.

We integrated the results from all analyses and were able to construct a comprehensive network, consisting of 86 nodes, including 36 SNPs and 50 transcripts, and 115 lines, including 12 lines representing 4 pairs of epistasis genes (Fig. 1). There were two types of associations in the network. 22 QTSs and 29 QTTs were directly associated with the startle phenotype, while 39 tQTTs and 14 tQTSs were indirectly associated with the startle phenotype. The widths of the lines indicated the relative value of estimated genetic effects, in which a wider line showed a higher estimated genetic effect for the association and a slimmer line *per contra*. Epistasis existed at QTS and tQTS levels (Fig. 1). Epistasis effects were much smaller than main QTT effects, and could have opposite effects with different loci. GO analysis showed that *CG4982* is involved in neurogenesis.

Finally, we searched interactions between homologous genes using *BioPubInfo* (http://ibi.zju.edu.cn/biopubinfo/) to construct a regulatory network similar patterns shown in *Drosophila* (Figs S1–S4). The homologous genes corresponding to QTSs, QTTs, tQTTs, and tQTSs were presented as red nodes. In Figure S1, gene *GRPEL1* associated with 14 genes, and gene *NADK* was associated with five genes (four were shared with *GRPEL1*). Genes *RHBDL3*, *MITD1* and *SLC2A8* were jointly associated with function of membrane. Genes *NADK* and *EFTUD1* were jointly associated with function of nucleotide binding. In Figure S2, four genes of QTTs (*DNAH5*, *TRMU*, *ABCC9* and *MAP4K3*) were jointly associated with function of ATP binding. In Figure S3, five genes of tQTTs were associated with 13 genes by protein-protein interactions shown in dark blue lines. These interactions indicated that they participated in common pathways and were involved in the same cellular processes in the form of protein complex.

## Discussion

Recent results of human GWAS have explained relatively small heritability for many complex traits caused by missing heritability^28^. In this study, the total heritability was 93.48% in QTS mapping, and 99.63% in QTT mapping. In addition, the heritability for each transcript in tQTTs was high on average (Table S3). It was considered that additive effects were important genetic components for variation of startle behavior traits. Additive effects accounted for most QTSs, while previous studies showed that epistasis effects were very common in *Drosophila* ^29,30^. Gene *aop* was involved in regulation of nervous system development, neuron projection development and positive regulation of neuron differentiation. Gene *glu* played an important role in peripheral nervous system development. In QTTs, sex-specific effects contributed a small portion (0.49%) to the total heritability, while individual loci effects accounted for the most part (99.14%). Sex difference may result from different courtship patterns^31^. Gene *Hsp70Bc* was involved in stress response and response to abiotic stimulus, while gene *Gr22f* was associated with functions like neurological system process and sensory perception^27^.

Previous GWAS on startle behavior of *Drosophila* revealed dozens of candidate genes^21^, but in present study, due to the development of new methods and availability for analyzing larger biological data sets, various intermediate endophenotypes can be interrogated via association studies to identify mechanistic links between genotypic variation and gene expression^32^. Compared with previous studies on startle response, we replicated five genes that are significantly associated with startle response and firstly integrated SNP and transcript data together to construct a comprehensive genetic network, elaborating how some SNPs and transcripts affected phenotype via transcript. Association mapping of tQTSs and tQTTs can provide insight into the genetic architecture of variation in gene expression. Genes encoding proteins participating in the same pathway or being members of the same protein complex are often co-regulated^33^. Thus, several tQTTs can regulate more than one QTT and some tQTTs were also controlled by another tQTT (Fig. 1); they may act together to regulate downstream targets. This is important for revealing the genetic basis underlying complex traits, and enables researchers to investigate the genetic architecture from a higher perspective.

Our study showed that a large fraction of the genome and transcriptome was associated with phenotypic variation in startle-induced locomotion. The effects of most genes were small and many of them have pleiotropic effects. Integration of our data revealed a complex network of genes including transcriptional regulators^2^. Combinatorial regulation, the tendency of two or more regulators controlling the same target, plays an essential role in transcriptional regulation^34^, and this tendency is also revealed by our results (Fig. 1). Such integrated network structure has advantageous from an evolutionary perspective, as it buffers the effects of strong loss-of-function mutations^33^. Indeed, phenotypic robustness, even as the organism is faced with genetic or environmental perturbations, is commonly attributed to features of underlying networks, such as gene connectivity, co-expression relations and the presence of microRNAs^11^. Understanding the nature of phenotypic robustness is central to predictions of individual genetic risk to disease, and the ability to select superior lines in animal and plant breeding^35^.

Similar to *Drosophila*, the corresponding human homologous gene network shows exists pervasive epistasis effects. For example, genes *RFC5* and *CRYAB* are associated with *DPP4* by protein binding (Figure S4). In addition, genes *RfC3* (corresponding to *RFC5*) and *l(2)efl* (corresponding to *CRYAB*) both have additive effects with *CG11034* (corresponding to *DPP4*; Fig. 1). Information on context-dependent effects in humans is sparse. However, evolutionary conservation of key genes and pathways between flies and humans enable insights obtained from *Drosophila* to serve as a guide for studies of human genetics^36^, and a good approach to explore human complex diseases^37^.

## Electronic supplementary material

Supplementary Information
