# Supplementary material for: Dissecting genetic architecture of startle response in Drosophila melanogaster using multi-omics information
Source: Sci Rep. 2017 Sep 28;7:12367. doi: 10.1038/s41598-017-11676-1 (PMC5620086; doi:10.1038/s41598-017-11676-1)
Supplement: Supplementary file 1 — Supplementary Information [file 41598_2017_11676_MOESM1_ESM.doc]

# Dissecting the genetic architecture of startle response in *Drosophila melanogaster* using multi-omics information

# Angli Xue, Hongcheng Wang, Jun Zhu^*^

Institute of Bioinformatics, Zhejiang University, Hangzhou 310058, China

*** Correspondence to:**

Professor Jun Zhu

Institute of Bioinformatics

Zhejiang University

Hangzhou, Zhejiang, China

Tel: (571) 88982731

Email: [jzhu@zju.edu.cn](mailto:jzhu@zju.edu.cn)

**Table S1**. Predicted effects of QTSs associated with startle response in *Drosophila melanogaster*

| Chr_Locus_Allele | SNP ID | Gene | Human Homolog | Effect | Estimate | SE | −Log*P_EW_* | *h*^2^(%) |
| --- | --- | --- | --- | --- | --- | --- | --- | --- |
| 2L_2166044_G | rs204115446 | *aop* | */* | *a* | 0.400 | 0.089 | 5.1 | 0.88 |
| 2L_4249496_T | rs207172383 | */* | */* | *a* | 0.912 | 0.088 | 24.3 | 4.56 |
| 2L_9318698_T | rs203338267 | */* | */* | *a* | -0.573 | 0.093 | 9.2 | 1.80 |
| 2L_9618432_T | rs205089822 | *GlcAT-S* | */* | *a* | -0.589 | 0.100 | 8.4 | 1.90 |
| 2L_9946120_A | rs202248051 | *CG5853* | */* | *a* | 0.408 | 0.090 | 5.2 | 0.91 |
| 2L_10179297_C | rs205437221 | */* | */* | *a* | 0.472 | 0.093 | 6.4 | 1.22 |
| 2L_12568798_C | rs202613659 | */* | */* | *a* | -1.021 | 0.094 | 26.7 | 5.71 |
| 2L_16737240_G | rs204830415 | *ChLD3/glu* | */* | *a* | 0.610 | 0.091 | 10.6 | 2.04 |
| 2L_16955558_G | rs204578851 | */* | */* | *a* | -0.838 | 0.090 | 19.8 | 3.85 |
| 2L_18140472_G | rs203845085 | */* | */* | *a* | 0.470 | 0.092 | 6.6 | 1.21 |
| 2L_21053780_T | rs205850013 | *CG42238/betaInt-nu* | */* | *a* | -0.709 | 0.091 | 14.3 | 2.76 |
| 2R_1441701_T | rs204264647 | */* | */* | *a* | -1.250 | 0.150 | 16.1 | 8.57 |
| 2R_3371083_C | rs203919987 | *wech* | *TRIM71* | *a* | -0.550 | 0.091 | 8.8 | 1.66 |
| 2R_6832586_T | rs206433552 | */* | */* | *a* | -0.430 | 0.090 | 5.7 | 1.01 |
| 2R_7564258_T | rs206186809 | *Roc2/Tret1-2* | */SLC2A8* | *a* | 0.785 | 0.089 | 17.8 | 3.38 |
| 2R_8992604_C | rs207025574 | */* | */* | *a* | -0.422 | 0.089 | 5.7 | 0.97 |
| 2R_9440481_A | rs202355372 | *CG33156/Roe1* | *NADK/GRPEL1* | *a* | -0.644 | 0.090 | 12.2 | 2.27 |
| 2R_9492605_G | rs203339984 | */* | */* | *a* | -0.592 | 0.089 | 10.5 | 1.92 |
| 2R_12478858_G | rs203413738 | *CG8306* | */* | *a* | -0.461 | 0.089 | 6.6 | 1.16 |
| 2R_13772190_A | rs205332615 | *elk* | */* | *a* | -0.932 | 0.090 | 24.5 | 4.76 |
| 2R_19331212_A | rs207355461 | */* | */* | *a* | 0.515 | 0.090 | 7.9 | 1.45 |
| 3L_1476862_C | rs204655088 | *robl62A/stet* | */RHBDL3* | *a* | -0.618 | 0.088 | 11.6 | 2.09 |
| 3L_4003193_T | rs202989040 | *CG14985* | *MITD1* | *a* | -0.684 | 0.088 | 14.2 | 2.56 |
| 3L_5307119_T | rs205417279 | */* | */* | *a* | 0.504 | 0.094 | 7.1 | 1.39 |
| 3L_7620131_G | rs202620123 | *CG33275* | */* | *a* | -0.752 | 0.091 | 15.9 | 3.10 |
| 3L_9107394_T | rs204446321 | *bol* | */* | *a* | -0.529 | 0.088 | 8.7 | 1.54 |
| 3L_10705270_T | rs206316788 | */* | */* | *a* | 0.566 | 0.089 | 9.6 | 1.76 |
| 3L_12718576_C | rs205086474 | */* | */* | *a* | 0.621 | 0.093 | 10.5 | 2.11 |
| 3L_16483167_C | rs206127819 | *CG33158* | *EFTUD1* | *a* | 0.438 | 0.092 | 5.7 | 1.05 |
| 3L_16999840_C | rs206831862 | */* | */* | *a* | 0.582 | 0.091 | 9.8 | 1.85 |
| 3R_7635822_C | rs205656886 | *ClC-a* | */* | *a* | -0.477 | 0.092 | 6.7 | 1.25 |
| 3R_23764762_C | rs204508294 | *CG5514/Mes-4* | */* | *a* | 0.495 | 0.090 | 7.4 | 1.34 |
| 2L_4508285_T × 2L_12568798_C | rs205796138 × rs202613659 | *dp × /* | */* | *aa* | -0.467 | 0.096 | 5.9 | 2.39 |
| 2L_10179297_C × 2R_8992604_C | rs205437221 × rs207025574 | */* | */* | *aa* | 0.579 | 0.094 | 9.1 | 3.67 |
| 2L_12568798_C × 2L_18140472_G | rs202613659 × rs203845085 | */* | */* | *aa* | 0.662 | 0.098 | 10.8 | 4.80 |

Genetic effect: *a* = additive effect for QTS. *aa* = additive × additive epistasis effect for a pair of QTSs.

−Log*P_EW_* = minus Log_10_ (experiment-wise *P*-value). *h^2^* (%) = heritability (%).

**Table S2. Predicted effects of QTTs associated with startle response in *Drosophila melanogaster***

| Locus | Gene | Huamn Homolog | Effect | Estimate | SE | −Log*P_EW_* | *h^2^* (%) |
| --- | --- | --- | --- | --- | --- | --- | --- |
| FBgn0002567 | *Rab32* | *RAB32* | *q* | 2.495 | 0.099 | 136.5 | 1.10 |
| FBgn0002576 | *lz* | */* | *q* | -2.260 | 0.202 | 28.2 | 0.90 |
| FBgn0005670 | *Cyp4d1* | */* | *q* | -1.357 | 0.120 | 28.9 | 0.32 |
| FBgn0013279 | *Hsp70Bc* | *HSPA1A* | *q* | 0.564 | 0.104 | 7.2 | 0.06 |
| FBgn0015336 | *CG15865* | */* | *q* | 3.644 | 0.244 | 49.4 | 2.34 |
| FBgn0020376 | *Sr-CIII* | */* | *q* | -3.596 | 0.129 | 168.0 | 2.28 |
| FBgn0028675 | *CG5772* | *ABCC9* | *q* | 5.118 | 0.250 | 91.7 | 4.62 |
| FBgn0030108 | *Gr8a* | */* | *q* | -1.076 | 0.181 | 8.6 | 0.20 |
| FBgn0030327 | *FucT6* | *FUT8* | *q* | -1.571 | 0.218 | 12.2 | 0.44 |
| FBgn0031741 | *CG11034* | *DPP4* | *q* | -0.732 | 0.074 | 22.6 | 0.09 |
| FBgn0031815 | *frj* | *MBOAT7* | *q* | 1.735 | 0.188 | 19.6 | 0.53 |
| FBgn0031897 | *CG13784* | */* | *q* | 0.544 | 0.073 | 13.2 | 0.05 |
| FBgn0033645 | *CG13196* | */* | *q* | -4.941 | 0.173 | 174.6 | 4.30 |
| FBgn0034437 | *CG10051* | *ERMP1* | *q* | 2.607 | 0.214 | 33.4 | 1.20 |
| FBgn0034512 | *CG18067* | */* | *q* | -2.343 | 0.098 | 124.3 | 0.97 |
| FBgn0034683 | *CG13500* | */* | *q* | -1.640 | 0.144 | 29.2 | 0.47 |
| FBgn0036343 | *CG14115* | */* | *q* | -0.785 | 0.106 | 12.8 | 0.11 |
| FBgn0036977 | *CG5665* | */* | *q* | -2.871 | 0.187 | 52.0 | 1.45 |
| FBgn0037485 | *CG14606* | */* | *q* | -3.532 | 0.160 | 106.1 | 2.20 |
| FBgn0037726 | *CG9492* | *DNAH5* | *q* | -1.638 | 0.255 | 9.9 | 0.47 |
| FBgn0037923 | *CG6813* | */* | *q* | -4.065 | 0.156 | 147.0 | 2.91 |
| FBgn0038223 | *CG8538* | */* | *q* | 1.747 | 0.280 | 9.3 | 0.54 |
| FBgn0038348 | *CG18519* | */* | *q* | 4.898 | 0.241 | 90.3 | 4.23 |
| FBgn0040337 | *CG3021* | *TRMU* | *q* | -3.716 | 0.216 | 64.9 | 2.43 |
| FBgn0040959 | *CG17814* | */* | *q* | 2.354 | 0.076 | 203.8 | 0.98 |
| FBgn0041249 | *Gr22f* | */* | *q* | -1.892 | 0.128 | 48.7 | 0.63 |
| FBgn0050088 | *CG30088* | */* | *q* | 1.859 | 0.154 | 32.6 | 0.61 |
| FBgn0050272 | *CG30272* | */* | *q* | -3.089 | 0.172 | 70.5 | 1.68 |
| FBgn0051800 | *CG31800* | *C1orf50* | *q* | -1.924 | 0.096 | 88.2 | 0.65 |
| FBgn0053630 | *CG12808* | */* | *q* | 1.653 | 0.131 | 35.9 | 0.48 |
| FBgn0053635 | *CG33635* | *SFT2D3* | *q* | -2.782 | 0.114 | 129.3 | 1.36 |
| FBgn0053922 | *CG33922* | */* | *q* | 2.192 | 0.112 | 83.8 | 0.85 |
| FBgn0062412 | *Ctr1B* | */* | *q* | -1.417 | 0.077 | 74.5 | 0.35 |
| FBgn0085405 | *CG13845* | */* | *q* | 1.050 | 0.138 | 13.6 | 0.19 |
| FBgn0260482 | *CG32599* | */* | *q* | -3.086 | 0.179 | 65.6 | 1.68 |
| FBgn0261401 | *CG14586* | */* | *q* | 2.932 | 0.178 | 59.9 | 1.52 |
| FBgn0262534 | *CG43088* | */* | *q* | -1.696 | 0.179 | 20.5 | 0.51 |
| FBgn0263107 | *miR-263* | */* | *q* | -1.270 | 0.058 | 105.1 | 0.28 |
| FBgn0263395 | *hppy* | *MAP4K3* | *q* | -4.710 | 0.288 | 59.0 | 3.91 |
| FBgn0263548 | *mir-4957* | */* | *q* | -0.710 | 0.056 | 35.6 | 0.09 |
| FBgn0263582 | *CR43607* | */* | *q* | 0.602 | 0.090 | 10.7 | 0.06 |
| FBgn0264361 | *CG43813* | */* | *q* | -1.391 | 0.090 | 53.0 | 0.34 |
| FBgn0264987 | *CR44138* | */* | *q* | -0.990 | 0.085 | 30.6 | 0.17 |
| XLOC_000688 | */* | */* | *q* | 3.048 | 0.234 | 38.0 | 1.64 |
| XLOC_000792 | */* | */* | *q* | 3.686 | 0.182 | 90.0 | 2.40 |
| XLOC_000945 | */* | */* | *q* | -1.367 | 0.220 | 9.3 | 0.33 |
| XLOC_001023 | */* | */* | *q* | 2.137 | 0.193 | 27.6 | 0.81 |
| XLOC_001203 | */* | */* | *q* | -1.846 | 0.114 | 57.8 | 0.60 |
| XLOC_001473 | */* | */* | *q* | -3.860 | 0.196 | 85.0 | 2.63 |
| XLOC_001741 | */* | */* | *q* | -0.948 | 0.177 | 7.1 | 0.16 |
| XLOC_001779 | */* | */* | *q* | 2.253 | 0.168 | 40.2 | 0.90 |
| XLOC_001792 | */* | */* | *q* | 1.873 | 0.133 | 44.3 | 0.62 |
| XLOC_002172 | */* | */* | *q* | 2.245 | 0.116 | 81.4 | 0.89 |
| XLOC_002200 | */* | */* | *q* | -2.212 | 0.207 | 25.8 | 0.86 |
| XLOC_002224 | */* | */* | *q* | 3.529 | 0.167 | 97.6 | 2.20 |
| XLOC_002327 | */* | */* | *q* | -3.091 | 0.115 | 156.8 | 1.68 |
| XLOC_002412 | */* | */* | *q* | -1.369 | 0.165 | 16.0 | 0.33 |
| XLOC_002771 | */* | */* | *q* | -1.628 | 0.109 | 49.3 | 0.47 |
| XLOC_002803 | */* | */* | *q* | -1.720 | 0.121 | 44.8 | 0.52 |
| XLOC_002997 | */* | */* | *q* | 1.803 | 0.089 | 89.7 | 0.57 |
| XLOC_003032 | */* | */* | *q* | -1.962 | 0.159 | 34.0 | 0.68 |
| XLOC_003155 | */* | */* | *q* | 2.526 | 0.162 | 53.7 | 1.12 |
| XLOC_003194 | */* | */* | *q* | 3.345 | 0.258 | 37.6 | 1.97 |
| XLOC_003311 | */* | */* | *q* | -2.399 | 0.186 | 37.1 | 1.01 |
| XLOC_003424 | */* | */* | *q* | 3.899 | 0.160 | 128.3 | 2.68 |
| XLOC_003474 | */* | */* | *q* | 4.572 | 0.171 | 154.5 | 3.68 |
| XLOC_003539 | */* | */* | *q* | -5.505 | 0.184 | 191.6 | 5.34 |
| XLOC_003664 | */* | */* | *q* | 1.056 | 0.100 | 25.5 | 0.20 |
| XLOC_003673 | */* | */* | *q* | -3.671 | 0.190 | 81.9 | 2.38 |
| XLOC_003773 | */* | */* | *q* | 1.573 | 0.185 | 16.7 | 0.44 |
| XLOC_003859 | */* | */* | *q* | 1.011 | 0.138 | 12.6 | 0.18 |
| XLOC_004376 | */* | */* | *q* | -2.179 | 0.090 | 127.1 | 0.84 |
| XLOC_004899 | */* | */* | *q* | -1.237 | 0.100 | 34.5 | 0.27 |
| XLOC_005059 | */* | */* | *q* | -1.951 | 0.100 | 84.2 | 0.67 |
| XLOC_005107 | */* | */* | *q* | -0.313 | 0.051 | 9.0 | 0.02 |
| XLOC_005268 | */* | */* | *q* | 4.587 | 0.207 | 107.1 | 3.71 |
| XLOC_005360 | */* | */* | *q* | -3.438 | 0.205 | 62.2 | 2.08 |
| XLOC_005772 | */* | */* | *q* | 0.787 | 0.136 | 8.2 | 0.11 |
| XLOC_006106 | */* | */* | *q* | -2.821 | 0.122 | 116.9 | 1.40 |
| XLOC_006171 | */* | */* | *q* | -0.555 | 0.089 | 9.3 | 0.05 |
| XLOC_006232 | */* | */* | *q* | 3.348 | 0.106 | 214.4 | 1.98 |

Genetic effect: *q* = additive expression effect for QTT.

−Log*P* = minus Log_10_(experiment-wise *P*-value). *h^2^* (%) = heritability (%).

| **Table S3**. Predicted effects of tQTTs associated with QTTs controlling startle response in *Drosophila melanogaster* | | | | | | | | | |  |
| --- | --- | --- | --- | --- | --- | --- | --- | --- | --- | --- |
| QTT | Gene | Human homolog | tQTT | Gene | Human homolog | Effect | Estimate | SE | −Log*P_EW_* | *h*^2^(%) |
| FBGN0002567 | *Rab32* | *RAB32* | FBgn0031836 | *CG11050* | *HDDC2* | *q* | 0.266 | 0.069 | 4.0 | 15.69 |
|  |  |  | FBgn0250789 | *alpha-Spec* | *SPTAN1* | *q* | 0.247 | 0.062 | 4.2 | 13.60 |
|  |  |  | XLOC_003577 | */* | */* | *qe_1_* | -0.276 | 0.070 | 4.1 | 16.96 |
|  |  |  |  |  |  | *qe_2_* | 0.274 | 0.070 | 4.0 | 16.65 |
| FBGN0005670 | *Cyp4d1* | */* | FBgn0030148 | *CG3106* | */* | *q* | 0.130 | 0.031 | 4.5 | 5.08 |
|  |  |  | FBgn0034436 | *CG11961* | *ERMP1* | *q* | 0.227 | 0.043 | 6.8 | 15.43 |
| FBGN0013279 | *Hsp70Bc* | *HSPA1A* | FBgn0036459 | *CG3349* | */* | *q* | 0.289 | 0.070 | 4.5 | 13.46 |
|  |  |  | FBgn0040813 | *Nplp2* | */* | *q* | -0.265 | 0.063 | 4.6 | 11.35 |
|  |  |  | XLOC_004723 | */* | */* | *q* | 0.225 | 0.061 | 3.6 | 8.19 |
| FBGN0020376 | *Sr-CIII* | */* | FBgn0047092 | *CR32730* | */* | *q* | 0.253 | 0.069 | 3.6 | 15.05 |
| FBGN0028675 | *CG5772* | *ABCC9* | FBgn0031619 | *CG3355* | *TMPRSS3* | *q* | 0.086 | 0.024 | 3.4 | 6.40 |
|  |  |  | FBgn0032290 | *CG6443* | *RTFDC1* | *q* | -0.074 | 0.018 | 4.3 | 4.84 |
|  |  |  | FBgn0051800 | *CG31800* | *C1orf50* | *q* | 0.101 | 0.014 | 12.2 | 8.99 |
|  |  |  | FBgn0052638 | *CG32638* | */* | *q* | -0.096 | 0.024 | 4.1 | 8.02 |
|  |  |  | XLOC_006377 | */* | */* | *q* | 0.184 | 0.031 | 8.7 | 29.61 |
| FBGN0030327 | *FucT6* | *FUT8* | FBgn0010772 | *Xe7* | */* | *q* | 0.134 | 0.039 | 3.3 | 20.41 |
|  |  |  | FBgn0262684 | */* | */* | *q* | 0.152 | 0.044 | 3.3 | 26.44 |
| FBGN0031741 | *CG11034* | *DPP4* | FBgn0035022 | *CG11413* | */* | *q* | 0.418 | 0.105 | 4.2 | 13.45 |
|  |  |  | FBgn0035360 | *CG1246* | */* | *q* | 0.228 | 0.067 | 3.2 | 4.00 |
|  |  |  | FBgn0262297 | *mir-2496* | */* | *q* | 0.235 | 0.046 | 6.4 | 4.25 |
|  |  |  | XLOC_003523 | */* | */* | *q* | 0.191 | 0.053 | 3.5 | 2.82 |
|  |  |  | XLOC_003687 | */* | */* | *q* | 0.283 | 0.076 | 3.7 | 6.16 |
| FBGN0031897 | *CG13784* | */* | FBgn0038292 | *CG3987* | */* | *q* | 0.181 | 0.049 | 3.7 | 4.65 |
| FBGN0033645 | *CG13196* | */* | FBgn0039214 | *puf* | *USP34* | *q* | -0.366 | 0.080 | 5.3 | 41.16 |
|  |  |  | FBgn0085225 | *CG34196* | */* | *q* | 0.078 | 0.021 | 3.7 | 1.89 |
|  |  |  | FBgn0263247 | *CG43390* | */* | *q* | 0.050 | 0.013 | 3.8 | 0.77 |
|  |  |  | FBgn0264838 | *CR44046* | */* | *q* | 0.057 | 0.013 | 4.6 | 0.99 |
|  |  |  | XLOC_000602 | */* | */* | *q* | 0.142 | 0.041 | 3.3 | 6.22 |
| FBGN0034437 | *CG10051* | *ERMP1* | FBgn0259176 | *bun* | */* | *q* | -0.162 | 0.046 | 3.4 | 23.91 |
| FBGN0034512 | *CG18067* | */* | FBgn0041182 | *Tep2* | */* | *q* | 0.227 | 0.065 | 3.4 | 9.11 |
| FBGN0037485 | *CG14606* | */* | FBgn0033608 | *CG13220* | */* | *q* | -0.150 | 0.038 | 4.0 | 9.85 |
|  |  |  | FBgn0051272 | *CG31272* | */* | *q* | 0.145 | 0.020 | 12.9 | 9.26 |
|  |  |  | XLOC_001023 | */* | */* | *q* | 0.132 | 0.039 | 3.1 | 7.64 |
|  |  |  | XLOC_004801 | */* | */* | *q* | -0.120 | 0.028 | 4.8 | 6.26 |
| FBGN0038348 | *CG18519* | */* | XLOC_002250 | */* | */* | *q* | 0.083 | 0.021 | 4.1 | 8.02 |
|  |  |  | XLOC_003694 | */* | */* | *q* | 0.097 | 0.022 | 5.2 | 10.88 |
| FBGN0040959 | *CG17814* | */* | FBgn0031611 | *CG17840* | *FIG4* | *q* | 0.500 | 0.138 | 3.5 | 25.73 |
|  |  |  | FBgn0035944 | *CG5021* | *TVP23A* | *q* | 0.289 | 0.081 | 3.5 | 8.58 |
|  |  |  | FBgn0036078 | *Or67c* | */* | *q* | 0.205 | 0.058 | 3.4 | 4.32 |
|  |  |  | FBgn0083001 | *CR34597* | */* | *q* | 0.086 | 0.022 | 4.1 | 0.75 |
|  |  |  | XLOC_003495 | */* | */* | *q* | 0.160 | 0.036 | 5.0 | 2.63 |
| FBGN0050088 | *CG30088* | */* | FBgn0262317 | *mir-966* | */* | *q* | 0.107 | 0.029 | 3.7 | 5.79 |
| FBGN0050272 | *CG30272* | */* | FBgn0031752 | *CG9044* | */* | *q* | -0.273 | 0.066 | 4.5 | 30.63 |
|  |  |  | FBgn0045478 | *Gr64b* | */* | *q* | 0.123 | 0.034 | 3.5 | 6.19 |
|  |  |  | XLOC_004948 | */* | */* | *q* | 0.145 | 0.040 | 3.5 | 8.61 |
| FBGN0051800 | *CG31800* | *C1orf50* | FBgn0028675 | *CG5772* | *ABCC9* | *q* | 0.624 | 0.092 | 11.0 | 57.82 |
| FBGN0261401 | *CG14586* | */* | FBgn0030484 | *GstT4* | */* | *q* | -0.152 | 0.027 | 7.9 | 17.74 |
| FBGN0264361 | *CG43813* | */* | FBgn0051028 | *CG31028* | */* | *q* | 0.360 | 0.106 | 3.2 | 22.97 |
| FBGN0264987 | *CR44138* | */* | XLOC_001785 | */* | */* | *q* | 0.250 | 0.067 | 3.7 | 8.02 |
| XLOC_000688 | */* | */* | FBgn0033932 | *Dh44-R1* | *CRHR2* | *q* | 0.101 | 0.028 | 3.5 | 5.11 |
|  |  |  | XLOC_000192 | */* | */* | *q* | 0.077 | 0.022 | 3.2 | 2.95 |
|  |  |  | XLOC_004512 | */* | */* | *q* | -0.244 | 0.041 | 8.4 | 29.99 |
|  |  |  | XLOC_006089 | */* | */* | *q* | 0.181 | 0.043 | 4.7 | 16.49 |
|  |  |  | XLOC_006189 | */* | */* | *q* | 0.053 | 0.013 | 4.5 | 1.43 |
| XLOC_000792 | */* | */* | FBgn0037584 | *CG7963* | */* | *q* | 0.119 | 0.030 | 4.2 | 9.81 |
|  |  |  | XLOC_003129 | */* | */* | *q* | 0.112 | 0.032 | 3.4 | 8.76 |
| XLOC_001023 | */* | */* | FBgn0027279 | *l(1)G0196* | *PPIP5K2* | *q* | -0.267 | 0.042 | 9.6 | 18.80 |
|  |  |  | FBgn0028490 | *CG31705* | */* | *q* | -0.086 | 0.019 | 5.4 | 1.94 |
|  |  |  | FBgn0031802 | *ppk7* | */* | *q* | 0.132 | 0.031 | 4.8 | 4.59 |
|  |  |  | FBgn0032442 | *CG15485* | */* | *q* | 0.134 | 0.037 | 3.6 | 4.71 |
|  |  |  | FBgn0033648 | *Ir48b* | */* | *q* | 0.168 | 0.036 | 5.6 | 7.40 |
|  |  |  | FBgn0036586 | *CG13070* | */* | *q* | 0.082 | 0.017 | 5.8 | 1.76 |
|  |  |  | FBgn0037098 | *Wnk* | */* | *q* | 0.168 | 0.044 | 3.8 | 7.44 |
|  |  |  | FBgn0085278 | */* | */* | *qe_1_* | 0.054 | 0.014 | 3.9 | 0.77 |
|  |  |  |  |  |  | *qe_2_* | -0.055 | 0.014 | 4.1 | 0.80 |
|  |  |  | FBgn0262951 | *CR43270* | */* | *qe_1_* | -0.196 | 0.059 | 3.1 | 10.11 |
|  |  |  |  |  |  | *qe_2_* | 0.200 | 0.059 | 3.2 | 10.49 |
|  |  |  | FBgn0263040 | *CG43335* | */* | *q* | 0.077 | 0.017 | 5.4 | 1.58 |
| XLOC_001473 | */* | */* | FBgn0261862 | *whd* | */* | *q* | -0.110 | 0.025 | 4.9 | 8.28 |
|  |  |  | XLOC_003346 | */* | */* | *q* | 0.157 | 0.038 | 4.4 | 16.88 |
| XLOC_001792 | */* | */* | FBgn0000658 | *fj* | *FJX1* | *q* | 0.257 | 0.054 | 5.6 | 19.17 |
|  |  |  | XLOC_003579 | */* | */* | *q* | 0.118 | 0.029 | 4.4 | 4.04 |
|  |  |  | XLOC_004820 | */* | */* | *q* | -0.236 | 0.047 | 6.4 | 16.18 |
| XLOC_002200 | */* | */* | XLOC_005110 | */* | */* | *q* | 0.118 | 0.028 | 4.7 | 9.47 |
| XLOC_002327 | */* | */* | FBgn0265095 | *CR44200* | */* | *q* | 0.096 | 0.026 | 3.6 | 2.31 |
| XLOC_002412 | */* | */* | FBgn0004895 | *fd64A* | */* | *q* | 0.140 | 0.038 | 3.6 | 10.04 |
|  |  |  | FBgn0034183 | *CG9642* | */* | *q* | 0.191 | 0.040 | 5.7 | 18.79 |
|  |  |  | FBgn0261833 | *CR42765* | */* | *qe_1_* | 0.125 | 0.035 | 3.5 | 8.05 |
|  |  |  |  |  |  | *qe_2_* | -0.126 | 0.035 | 3.5 | 8.20 |
|  |  |  | XLOC_003376 | */* | */* | *q* | 0.081 | 0.022 | 3.6 | 3.34 |
| XLOC_002997 | */* | */* | XLOC_001405 | */* | */* | *q* | 0.442 | 0.109 | 4.3 | 27.34 |
| XLOC_003032 | */* | */* | FBgn0053767 | *CG33767* | */* | *q* | 0.155 | 0.035 | 5.2 | 12.73 |
| XLOC_003194 | */* | */* | FBgn0041243 | *Gr43a* | */* | *q* | 0.075 | 0.022 | 3.2 | 5.72 |
| XLOC_003311 | */* | */* | XLOC_003686 | */* | */* | *q* | 0.135 | 0.030 | 5.2 | 13.79 |
| XLOC_003424 | */* | */* | FBgn0033932 | *Dh44-R1* | *CRHR2* | *q* | 0.155 | 0.038 | 4.2 | 11.14 |
| XLOC_003474 | */* | */* | FBgn0032400 | *CG6770* | */* | *q* | -0.174 | 0.035 | 6.3 | 16.47 |
|  |  |  | FBgn0036410 | *CG8100* | */* | *q* | 0.099 | 0.029 | 3.3 | 5.36 |
| XLOC_003539 | */* | */* | FBgn0032821 | *CdGAPr* | */* | *q* | -0.240 | 0.052 | 5.5 | 25.55 |
|  |  |  | FBgn0262680 | *CR43150* | */* | *q* | 0.069 | 0.018 | 3.8 | 2.08 |
|  |  |  | XLOC_000559 | */* | */* | *q* | 0.095 | 0.024 | 4.1 | 4.01 |
| XLOC_003664 | */* | */* | XLOC_003291 | */* | */* | *q* | 0.148 | 0.028 | 7.0 | 8.68 |
| XLOC_003773 | */* | */* | XLOC_006411 | */* | */* | *q* | 0.137 | 0.031 | 5.0 | 13.65 |
| XLOC_005059 | */* | */* | XLOC_000124 | *CR44490* | */* | *q* | 0.129 | 0.025 | 6.4 | 5.19 |
| XLOC_005107 | */* | */* | FBgn0011953 | *tRNA:R:12Ef* | */* | *q* | 0.151 | 0.034 | 5.2 | 2.08 |
| XLOC_005268 | */* | */* | FBgn0027279 | *l(1)G0196* | *PPIP5K2* | *q* | -0.143 | 0.042 | 3.2 | 18.73 |
|  |  |  | FBgn0036638 | *CG13033* | */* | *q* | 0.117 | 0.033 | 3.4 | 12.62 |
| XLOC_005772 | */* | */* | XLOC_000451 | */* | */* | *q* | 0.166 | 0.041 | 4.2 | 12.12 |

| Genetic effect: *q* = additive expression effect for tQTT, *qe_1_* = male specific additive effect, *qe_2_* = female specific additive effect.  −Log*P* = minus Log_10_(experiment-wise *P*-value). *h^2^*(%) = heritability (%). |  |  |  |  |  |
| --- | --- | --- | --- | --- | --- |

**Table S4**. Predicted effects of tQTSs associated with QTTs controlling startle response in *Drosophila melanogaster*

| QTT | Gene | Human homolog | tQTS | Gene | Human homolog | Effect | Estimate | −Log*P_EW_* | *h*^2^(%) |
| --- | --- | --- | --- | --- | --- | --- | --- | --- | --- |
| FBgn0031741 | *CG11034* | *DPP4* | 2L_10418534_T | *Klp31E/RfC3* | *KIF21A/RFC5* | *a* | 0.125 | 3.8 | 3.13 |
|  |  |  | 2L_12568798_C | */* | */* | *a* | 0.119 | 3.2 | 2.83 |
|  |  |  | 2R_1366164_A | *CG30440* | */* | *a* | 0.140 | 4.4 | 3.95 |
|  |  |  | 2R_19572054_G | *CG11293/CG5431/l(2)efl* | */ / CRYAB* | *a* | -0.117 | 3.5 | 2.75 |
|  |  |  | 3L_6952456_G | *CG10075/sgl* | *UQCC1/UGDH* | *a* | 0.130 | 4.0 | 3.41 |
|  |  |  | 3L_15762324_T | */* | */* | *a* | -0.188 | 7.5 | 7.08 |
|  |  |  | 3R_14819254_C | *gukh* | */* | *a* | 0.150 | 5.2 | 4.5 |
|  |  |  | 3R_15677301_C | *CG4662* | *MICU3* | *a* | 0.130 | 4.2 | 3.41 |
| FBgn0031815 | *frj* | *MBOAT7* | 2R_11446640_G | *CG12963* | */* | *a* | -0.043 | 4.0 | 4.25 |
|  |  |  | 3L_9107399_A | *bol* | */* | *a* | -0.066 | 8.6 | 9.82 |
| FBgn0034512 | *CG18067* | */* | 2L_7516966_A | *Rapgap1* | */* | *a* | 0.096 | 3.8 | 2.89 |
|  |  |  | 2L_13338686_A | */* | */* | *a* | 0.123 | 5.8 | 4.68 |
|  |  |  | 2R_8651434_C | *CG12374* | */* | *a* | -0.162 | 8.3 | 8.15 |
|  |  |  | 2R_13603136_T | *CG6424* | */* | *a* | -0.187 | 12.8 | 10.82 |
|  |  |  | 3R_9628269_T | */* | */* | *a* | 0.107 | 3.5 | 3.54 |
|  |  |  | 2R_13603136_T x 3L_16294325_G | *CG6424 × CG4982* | */* | *aa* | 0.123 | 5.6 | 9.37 |
| FBgn0040959 | *CG17814* | */* | 2L_9845842_G | *nAcRalpha-30D* | */* | *a* | -0.156 | 6.1 | 6.84 |
|  |  |  | 2L_11448052_A | */* | */* | *a* | 0.146 | 5.6 | 5.98 |
|  |  |  | 2L_1015888_A | */* | */* | *a* | 0.065 | 4.3 | 5.23 |
| FBgn0062412 | *Ctr1B* | */* | 2L_20034637_A | *sNPF* | *PRLHR* | *a* | -0.202 | 8.8 | 11.46 |

Genetic effect: *a* = additive expression effect for tQTS, *aa* = additive × additive epistasis effect for a pair of tQTSs. −Log*P* = minus Log_10_(experiment-wise *P*-value). *h^2^*(%) = heritability (%).

**Table S5**. P-value comparison for five genes replicated from previous studies

| SNP/Flybase ID | Gene | Association type | −log_10_*P*_EW_ | -log_10_(p-value) in Mackay et al., 2012 &  Huang, Massouras, et al., 2014 |
| --- | --- | --- | --- | --- |
| rs205089822 | *GlcAT-S* | QTS | 8.4 | 4.5 |
| FBgn0026239 | *gukh* | tQTS | 5.2 | 5.2 |
| FBgn0027279 | *l(1)G0196* | tQTT | 9.6 | 5.9 |
| FBgn0033645 | *CG13196* | tQTT | 5.3 | 5.2 |
| FBgn0031752 | *CG9044* | tQTT | 4.5 | 2.2 |

***Table S6.*** *Startle-associated genes and their functions in GO analysis in Drosophila melanogaster*

| Gene | Full name | Functions* |
| --- | --- | --- |
| *aop* | *anterior open* | regulation of nervous system development, neuron differentiation, neuron projection development,regulation of neuron differentiation, negative regulation of neuron differentiation, positive regulation of neuron differentiation, neuron development, cell morphogenesis involved in neuron differentiation, neuron projection morphogenesis, sensory organ development, regulation of transcription, negative regulation of transcription, dendrite development, dendrite morphogenesis |
| *glu* | *gluon* | peripheral nervous system development |
| *gukh* | *GUK-holder* | nerve terminal, neuron projection, axon, axon part, terminal button, synapse part, synapse, presynaptic membrane |
| *Gr22f* | *Gustatory receptor 22f* | neurological system process,sensory perception,sensory perception of chemical stimulus,sensory perception,sensory perception of chemical stimulus |
| *Gr8a* | *Gustatory receptor 8a* | sensory perception,sensory perception of chemical stimulus, detection of chemical stimulus, neurological system process, cognition, detection of stimulus involved in sensory perception, detection of chemical stimulus involved in sensory perception, sensory perception of taste, detection of chemical stimulus involved in sensory perception of taste, detection of stimulus |
| *nAcRalpha-30D* | *nicotinic Acetylcholine Receptor alpha 30D* | neurotransmitter receptor activity, neurotransmitter binding, Neurotransmitter-gated ion-channel transmembrane region, Neurotransmitter-gated ion-channel, Neurotransmitter-gated ion-channel ligand-binding, Neurotransmitter-gated ion-channel, synapse part, synapse, postsynaptic membrane |
| *sNPF* | *short neuropeptide F precursor* | neuropeptide signaling pathway, neuropeptide hormone activity, neuropeptide, regulation of response to external stimulus |
| *betaInt-nu* | *beta[nu] integrin* | chemosensory behavior |
| *elk* | *eag-like K[+] channel* | regulation of transcription |
| *Hsp70Bc* | *Heat-shock-protein-70Bc* | cellular response to stress, stress response, stress-induced protein, response to abiotic stimulus |
| *l(2)efl* | *lethal (2) essential for life* | response to abiotic stimulus, stress response |
| *bun* | *bunched* | peripheral nervous system development, sensory organ development, negative regulation of signal transduction, regulation of RNA metabolic process |
| *alpha-Spec* | *alpha Spectrin* | regulation of neurotransmitter levels, neurotransmitter transport,, synaptic transmission, neurotransmitter secretion, neuromuscular synaptic transmission, axonogenesis, axon guidance, neuron regulation of synaptic growth at neuromuscular junction, oocyte differentiation, axon choice point recognition, axon midline choice point recognition, transmission of nerve impulse, neuron differentiation, neuron projection development, regulation of neurological system process, positive regulation of neurological system process, long-term strengthening of neuromuscular junction, neuron development, cell morphogenesis involved in neuron differentiation, maintenance of presynaptic active zone structure, neuron projection morphogenesis, regulation of synapse structure and activity, regulation of synaptic transmission, positive regulation of synaptic transmission, regulation of synapse organization, neurological system process, regulation of nervous system development, regulation of synaptogenesis, regulation of transmission of nerve impulse, positive regulation of transmission of nerve impulse |
| *CdGAPr* | *GTPase-activating protein CdGAPr* | axonogenesis, axon guidance, neuron differentiation, neuron projection development, retinal ganglion cell axon guidance, neuron development, cell morphogenesis involved in neuron differentiation, neuron projection morphogenesis |
| *Gr43a* | *Gustatory receptor 43a* | sensory perception, sensory perception of chemical stimulus, neurological system process |
| *Gr64b* | *Gustatory receptor 64b* | sensory perception, sensory perception of chemical stimulus, neurological system process |
| *Or67c* | *Odorant receptor 67c* | sensory perception, sensory perception of chemical stimulus, sensory perception of smell, neurological system process, sensory transduction |
| *fj* | *four-jointed* | sensory organ development |
| *Nplp2* | *Neuropeptide-like precursor 2* | neuropeptide signaling pathway, neuropeptide hormone activity |
| *DH44-R1* | *Dmel_CG8422* | neuropeptide hormone activity, neuropeptide receptor activity, neurotransmitter receptor activity, neurotransmitter binding, neuropeptide binding |
| *CG31272* | *Dmel_CG31272* | synaptic vesicle, synaptic vesicle membrane, synapse part, synapse |
| *CG9642* | *Dmel_CG9642* | transcription repressor activity, transcription regulator activity |
| *FD64A* | *forkhead domain 64A* | transcription, regulation of transcription, DNA-dependent, regulation of transcription, regulation of RNA metabolic process |
| All the function information are provided by DAVID: <https://david.ncifcrf.gov/> | | |


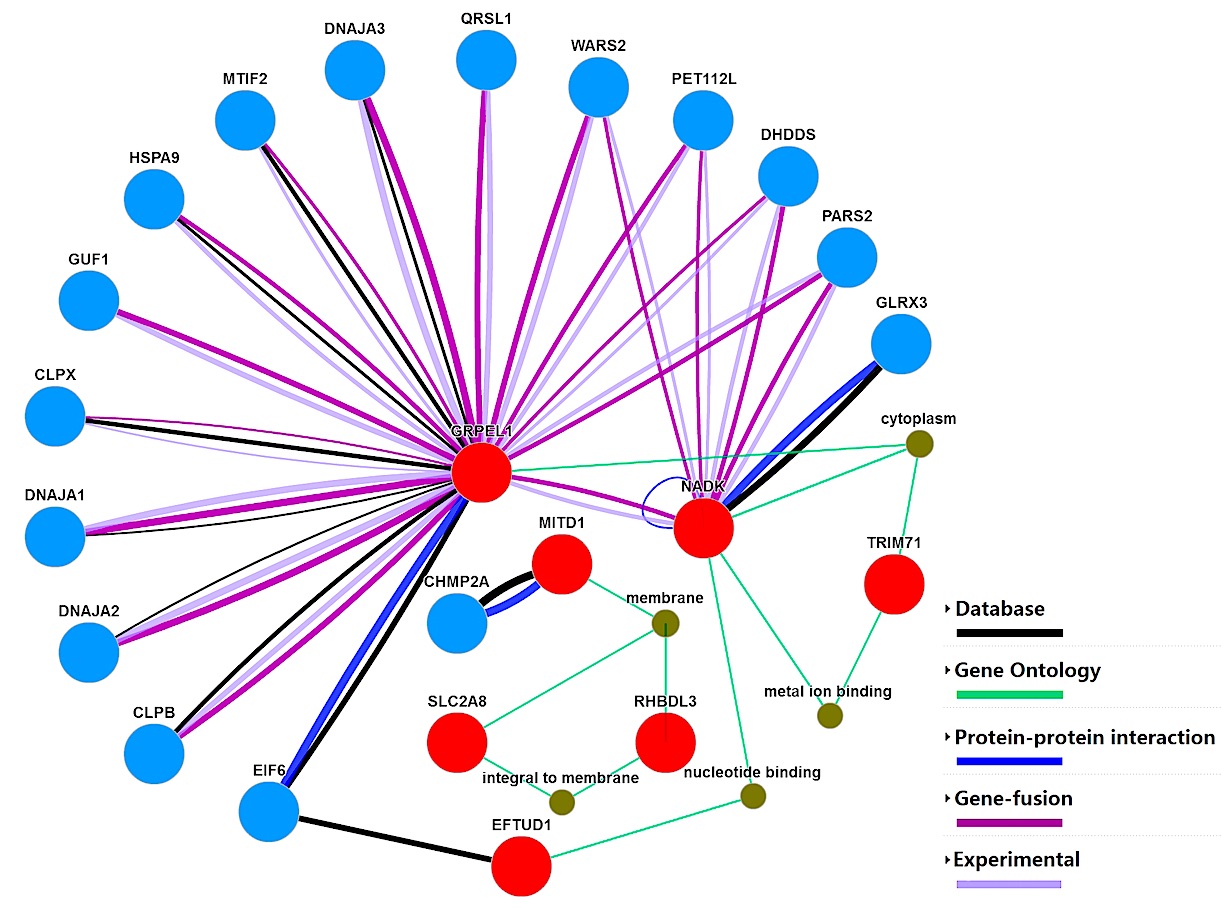


**Figure S1**. **A homologous network of corresponding human genes identified in QTSs.**

Notes: The size of nodes and thickness of lines stand for number of publications related. The red nodes denote homologous genes identified in QTSs. The olive nodes denote protein function, and the blue nodes denote association genes. Dark grey lines denote database, green lines denote the gene ontology, dark blue lines denote the protein-protein interactions, dark purple lines denote gene-fusion, and light purple lines denote experimental. The network shows extensive interactions between genome.


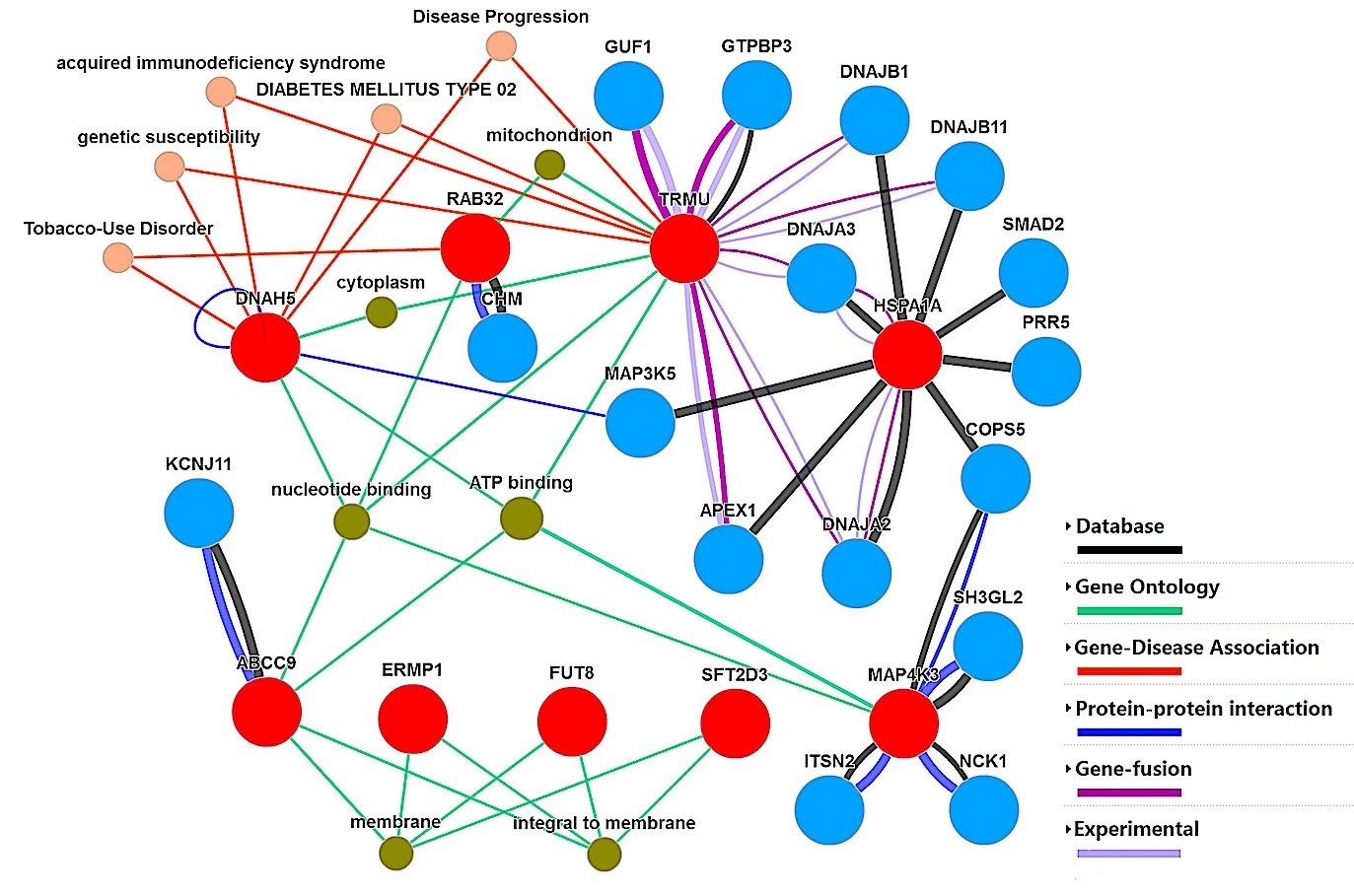
**Figure S2. A homologous network of corresponding human genes identified in QTTs.**

Notes: The size of nodes and thickness of lines stand for number of publications related. The red nodes denote homologous genes of transcripts identified in QTTs and tQTTs. The olive nodes denote protein function; the blue nodes denote association genes and orange nodes denote diseases and phenotype. Dark grey lines denote database, green lines denote the gene ontology, orange lines denote the gene-disease association and dark blue lines denote the protein-protein interaction. The network shows extensive interactions between transcriptome.


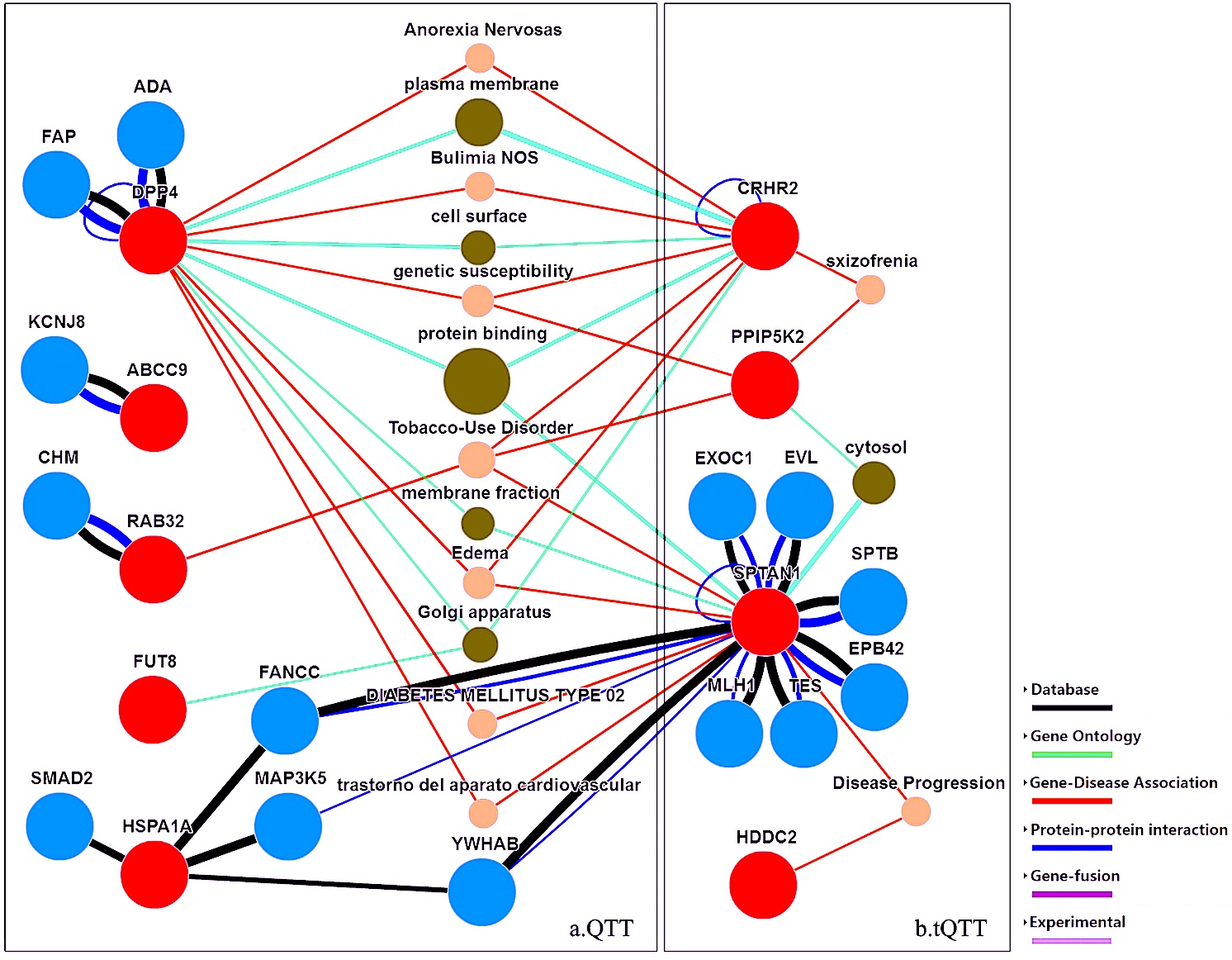
**Figure S3**. **A homologous network of corresponding human genes identified in QTTs/tQTTs.**

Notes: The size of nodes and thickness of lines stand for number of publications related. The red nodes denote homologous genes of transcripts identified in QTTs and tQTTs. The olive nodes denote protein function; the blue nodes denote association genes and orange nodes denote diseases and phenotype. Dark grey lines denote database, green lines denote the gene ontology, orange lines denote the gene-disease association, and dark blue lines denote the protein-protein interaction. The network shows extensive interactions between transcriptome.


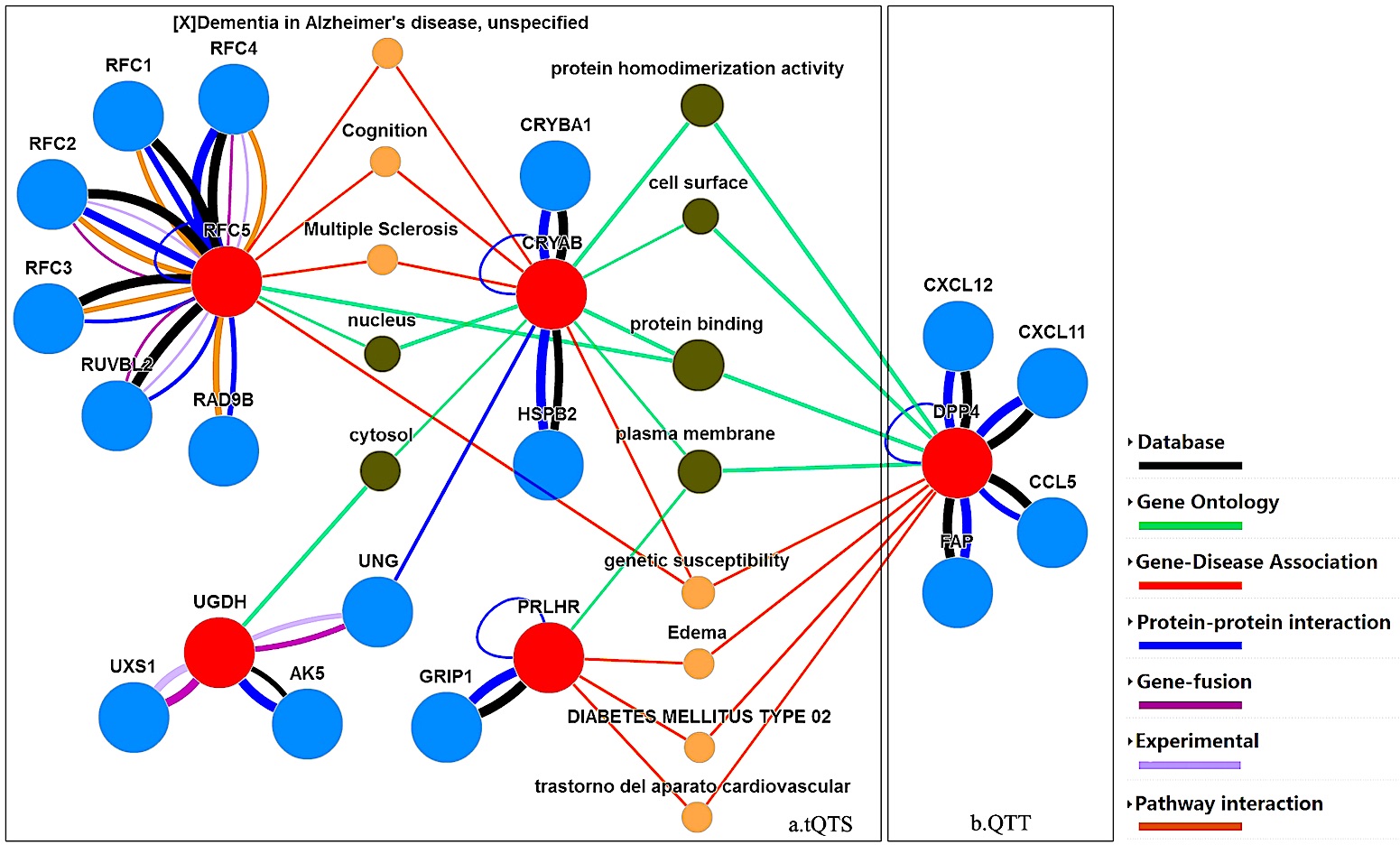


**Figure S4**. **A homologous network of corresponding human genes identified in QTTs/tQTSs.**

Notes: The size of nodes and thickness of lines stand for number of publications related. The red nodes denote homologous genes of transcripts identified in QTTs and tQTSs. The olive nodes denote protein function, the blue nodes denote association genes and orange nodes denote diseases and phenotype. Dark grey lines denote database, green lines denote the gene ontology, orange lines denote the gene-disease association, dark blue lines denote the protein-protein interaction and gold lines denote the pathway interaction. The network shows extensive interact.
